# Sarcopenia to frailty and malnutrition and their predictive role for postoperative outcomes in older gastric cancer

**DOI:** 10.1093/geroni/igaf122.2379

**Published:** 2025-12-31

**Authors:** Yinning Guo, Qin Xu, Ting Xu, Xinyi Xu

**Affiliations:** Nanjing Medical University, Nanjing, Jiangsu, China (People’s Republic); Nanjing Medical University, Nanjing, Jiangsu, China (People’s Republic); Nanjing Medical University, Nanjing, Jiangsu, China (People’s Republic); Nanjing Medical University, Nanjing, Jiangsu, China (People’s Republic)

## Abstract

**Objectives:**

This study aimed to investigate the prevalence of preoperative sarcopenia, frailty, and malnutrition in older gastric cancer patients, the correlation between sarcopenia and frailty as well as malnutrition, and the predictive role of them on postoperative outcomes.

**Methods:**

Older gastric cancer patients who were proposed to undergo radical surgery were selected and data were prospectively collected at admission and 3 months postoperatively. Regression models were used to explore the correlation between sarcopenia and frailty and malnutrition, as well as the predictive role of all three on functional quality, quality of life, and death at 3 months postoperatively. All models were adjusted.

**Results:**

624 patients were enrolled at admission and 595 completed follow-up 3 months after surgery. 46.3% patients with preoperative frailty, 44.7% with nutritional risk, and 9.5% with sarcopenia. There was a very strong correlation between sarcopenia and physical frailty, as well as nutritional risk and malnutrition(p<0.001). In terms of prediction, sarcopenia was a good predictor of quality of life and mortality at 3 months postoperatively, the three frailty subtypes of physical-social frailty, psychosocial frailty, and multidimensional frailty were good predictors of functional status, quality of life, and mortality, and malnutrition was effective in predicting quality of life.

**Conclusion:**

Sarcopenia was no less predictive of postoperative outcomes than malnutrition, but slightly less so than frailty. In the future, the interaction and joint predictive value of the three can be further analyzed to determine an objective, time-consuming, and predictive preoperative health risk assessment system for older cancer patients.

